# Global Prevalence and Device Related Causes of Needle Stick Injuries among Health Care Workers: A Systematic Review and Meta-Analysis

**DOI:** 10.5334/aogh.2698

**Published:** 2020-04-06

**Authors:** Salehoddin Bouya, Abbas Balouchi, Hosien Rafiemanesh, Mehrbanoo Amirshahi, Majid Dastres, Mahdieh Poodineh Moghadam, Niaz Behnamfar, Mahmood Shyeback, Mahin Badakhsh, Jasem Allahyari, Adhra Al Mawali, Abbas Ebadi, Asiyeh Dezhkam, Karen A. Daley

**Affiliations:** 1 Internal Medicine and Nephrology, Clinical Immunology Research Center, Ali-Ebne Abitaleb Hospital, Zahedan University of Medical Sciences, Zahedan, IR; 2 Student Research Committee, Nursing and Midwifery School, Iran University of Medical Sciences, Tehran, IR; 3 Student Research Committee, Department of Epidemiology, School of Public Health and Safety, Shahid Beheshti University of Medical Sciences, Tehran, IR; 4 Department of Midwifery, Zabol University of Medical Sciences, Zabol, IR; 5 Nursing and Midwifery school, Zahedan University of Medical Sciences, Zahedan, IR; 6 Department of Nursing, Faculty of Nursing and Midwifery, Zabol University of Medical Sciences, Zabol, IR; 7 Department of Nursing, Faculty of nursing and midwifery, Tehran medical sciences, Islamic Azad university, Tehran, IR; 8 Bushehr University of Medical Sciences, Bushehr, IR; 9 Zahedan University of medical sciences, Zahedan, IR; 10 Centre of Studies and Research, Oman Ministry of Health, Muscat, OM; 11 Behavioral Sciences Research Center, Life style institute, Baqiyatallah University of Medical Sciences, Tehran, IR; 12 Nursing Faculty, Baqiyatallah University of Medical Sciences, Tehran, IR; 13 Department of Pediaterics, Iranshahr University of Medical Sciences, Iranshahr, IR; 14 American Nurses Association, Massachusetts, US

## Abstract

**Background:**

Healthcare workers (HCWs) suffer more than 2 million occupational needle-stick injuries (NSIs) annually.

**Goal:**

To determine the global prevalence and causes of NSIs among HCWs.

**Methods:**

In this systematic review and meta-analysis, three databases (PubMed, Web of science, and Scopus) were searched for reports from January 1, 2000 to December 31, 2018. The random effects model was used to determine the prevalence of NSIs among HCWs. Hoy et al.’s instrument was employed to evaluate the quality of the included studies.

**Findings:**

A total of 87 studies performed on 50,916 HCWs in 31 countries worldwide were included in the study. The one-year global pooled prevalence of NSIs among HCWs was 44.5% (95% CI: 35.7, 53.2). Highest prevalence of NSIs occurred in the South East Asia region at 58.2% (95%, CI: 36.7, 79.8). By job category, prevalence of NSIs was highest among dentists at 59.1% (95% CI: 38.8, 79.4), Hypodermic needles were the most common cause of NSIs at 55.1% (95% CI: 41.4, 68.9).

**Conclusion:**

The current high prevalence of NSIs among HCWs suggests need to improve occupational health services and needle-stick education programs globally.

## Introduction

Currently, needlestick injuries (NSIs) are one of the most important occupational hazards among healthcare workers (HCWs) globally. According to WHO, more than two million occupational exposures to sharp injuries occur among 35 million HCWs annually [1].

NSIs increase the risk of over 20 types of infectious diseases among HCWs, including hepatitis B, hepatitis C, and HIV [2]. According to the Centers for Disease Control and Prevention (CDC) and European Agency for Safety and Health at Work (EU-OSHA) reports, there are more than 385,000 and 1,000,000 NSIs cases annually among hospital HCWs in the United States and Europe, respectively [3,4]. WHO statistics also show that NSIs cause 16,000, 66,000, and 1,000 cases of HCV, HBV, and HIV per year among HCWs, respectively [5]. The prevalence of various infectious diseases due to NSIs among HCWs is not a single and integrated phenomenon, rather is affected by several factors, such as vaccination rates among HCWs, access to appropriate worker protection equipment and post exposure prophylaxis (PEP), and compliance with precautionary infection control standards [6]. Additionally, the prevalence of NSIs is not the same among all HCWs, and NSIs occur more frequently among nurses, surgeons, and emergency personnel [7,8].

Each NSI case imposes a direct and indirect cost of 175 to 350 USD to the health care system [9]. NSI prevention is very important among HCWs. The first step in planning to prevent NSIs is to determine its precise prevalence rate, which is difficult due to a range of factors including predominantly voluntary reporting, lack of common denominators, lack of national surveillance systems, and suspected frequency of injury underreporting. Despite the importance of this issue, and in spite of individual studies, there are currently no accurate statistics on the global prevalence of NSIs among HCWs, especially in developing and less developed countries. Existing statistics are mainly published at the national level and are relevant to a limited number of developed countries. A national study conducted in the United States in 2017 referred to NSI as the main cause of percutaneous injuries in more than 71% of reported cases among HCWs [10]. Moreover, the results of annual surveys, even in developed countries such as the United States, have shown that despite the different strategies implemented, there is still an increasing incidence of NSIs among HCWs [11].

Determining the global prevalence and causes of NSIs may enable NSI rate reduction, creation of safer work environments and safety cultures, reduced turnover rate, reduced costs, and ultimately, provision of higher quality services among HCWs [12,13,14]. Previous reviews have examined the prevalence of NSIs only in a specific ward or only at the national level (Pakistan and Iran), or have investigated needlestick-related prevention and cost burden dimensions [9,15,16,17]. To the best of our knowledge, there has been no specific study on the global prevalence of NSIs so far. The aim of this study was to determine the global prevalence and device related causes of NSIs among HCWs.

## Methods

### Registration and Eligibility Criteria

The study protocol has been registered in International Prospective Register of Systematic Reviews, known as PROSPERO (CRD42019131562). This study was conducted using Cochrane guideline and reported using the Preferred Reporting Items for Systematic Reviews and Meta-Analyses (PRISMA) statement (Supplementary Table 1) [18].

Observational studies (cross-sectional) published in peer-reviewed journals from January 1, 2000 to December 31, 2018, conducted on healthcare workers, carried out on at least 25 people, were included in the study. Studies that aimed to determine the prevalence and causes of NSIs in at least one healthcare group over the past year were included [1]. The latest search on databases was performed on January 1, 2019. HCWs in this study include all employees who work in the health care system and are exposed to NSIs. HCWs included physicians, nurses, nursing and medical students, and other members of the health team. Exclusion criteria included studies published in non-English language before 2000, studies that reviewed the prevalence of needle stick among patients and other populations. As well as the studies that reported the prevalence of needle stick at other time periods, including last three months, or six months, or lifetime. Reviews, letters to the editor, high risk studies, short reports as well non-full text studies were also excluded.

### Search strategy

Three databases (PubMed, Web of science, and Scopus) were searched from January 1, 2000 to December 31, 2018. The PubMed search strategy was adopted to search in other databases. The search strategy was developed with the help of an experienced librarian in the field of systematic review studies in the health field. A combination of boolean operators (AND, OR, and NOT), Medical Subject Headings (MeSH), truncation “*”, Emtree and related keywords were used to search the articles. Keywords of the search included: Prevalence OR “Needlestick Injury” OR “sharp injuries” OR “health care workers” OR “health professionals” (Supplementary Table 2).

### Selection of studies, data extraction and quality assessment

Two researchers independently conducted search, screening, selection, and extraction of study data according to the study protocol. Disagreements were resolved via consensus method. After removing duplicates, the titles of the remaining articles were checked based on the purpose of the study and inclusion criteria. Then the abstract of the articles was examined and non-relevant items were excluded. Finally, the full-texts of included articles was reviewed. Then the required data was extracted in Microsoft Excel based on the study extraction form. The extracted data included: First Author, Year of publication, Country, Region (based on WHO categories in six group [African, Americans], EMRO [Eastern Mediterranean], European, South-East Asia, Western Pacific), Socio-demographic Index (SDI) status based on World Bank categories in four groups (high SDI, high middle SDI, low middle SDI, and low SDI), study period, sampling method, target population, type of study setting, participants, gender, prevalence of NSI/SI, causes of NSI/SI, and Risk of bias. The Hoy tool, specially designed for observational studies, was used to assess the quality of the studies included [19]. This 10-item tool evaluated the quality of studies in two dimensions including external validity (items 1–4 assess target population, sampling method and nonresponse bias minimal) and internal validity (items 5–9 assess data collection method, case definition, study instrument, mode of data collection and item 10 assesses bias related to the analysis).

### Data synthesis

We have reported all the retrieved data on global prevalence of NSIs in different groups, based on job categories of HCWs, WHO regions, country, and SDI. A random effects meta-analysis was conducted to determine pooled one-year prevalence estimates (with 95% confidence intervals [Cis]) of NSIs among HCWs. Also, sub-groups and meta-regression analyses were conducted to determine heterogeneity. The heterogeneity of the preliminary studies was evaluated using I^2^ tests, which determine the percentage of variation between studies due to heterogeneity, rather than by chance. In addition, meta-regression analyses were conducted for describing the linear relationship between (both continuous and categorical) study-level covariates and the prevalence of NSIs. Meta-analysis was performed using STATA 14 (StataCorp, Texas, USA) statistical software.

## Results

### Study selection

**Figure 1:**
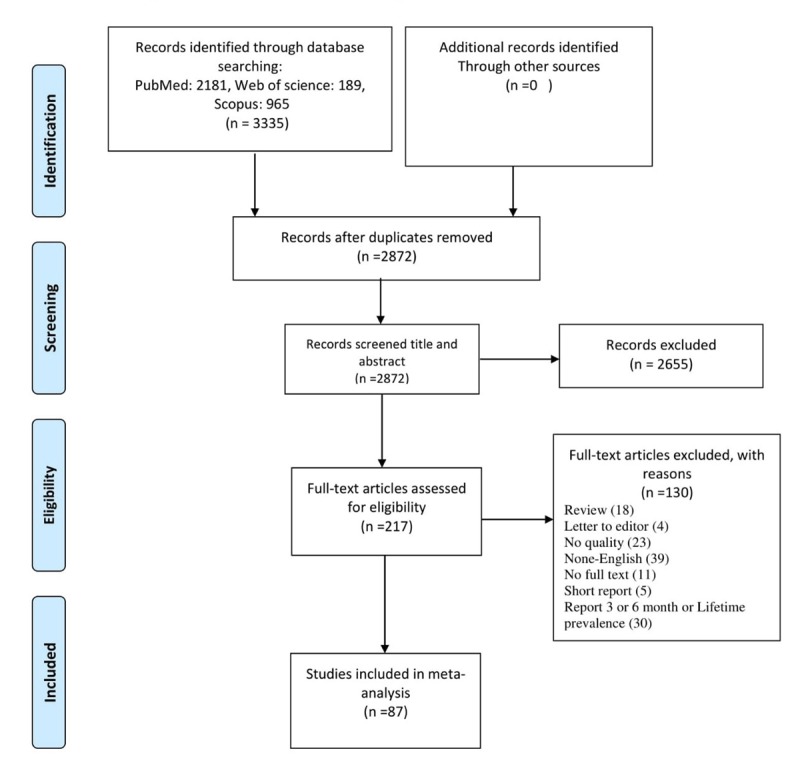
Study flow diagram.

A total of 3335 studies were retrieved from searches in three databases. Out of 2872 non-duplicated studies in the title and summary screening process, 2655 studies were excluded due to unrelated titles and abstracts. Out of 217 studies, 87 met eligibility criteria. Out of 130 excluded studies, 18 studies were reviews, four studies were letters to the editor, five studies were brief reports, 11 studies have no full text, 39 studies were published in non-English language, and 23 studies did not meet the minimum overall quality requirements for inclusion in the study. Also, 30 articles were excluded from the study since they reported the prevalence rate as life-time, 3, or 6 months (Figure 1).

### Study characteristics

A total of 87 studies performed on 50,916 HCWs in 31 countries from 2000 to 2018 were entered into the meta-analysis. Most studies were conducted in the Eastern Mediterranean region (EMRO) (n = 28) and western pacific regions (n = 20). Most studies were conducted in countries with middle to high SIDs (N = 65). The studies lasted anywhere between one day and 19 months. Most studies used census sampling (N = 34). Among HCWs, nurses were studied in most studies (n = 50). Moreover, most studies were multicenter research studies (n = 53). Of the 50,916 participants, most were nurses (n = 28371). All of the studies included had low risk of bias (Supplementary Table 3).

### Global prevalence of NSIs among HCWs

**Figure 2:**
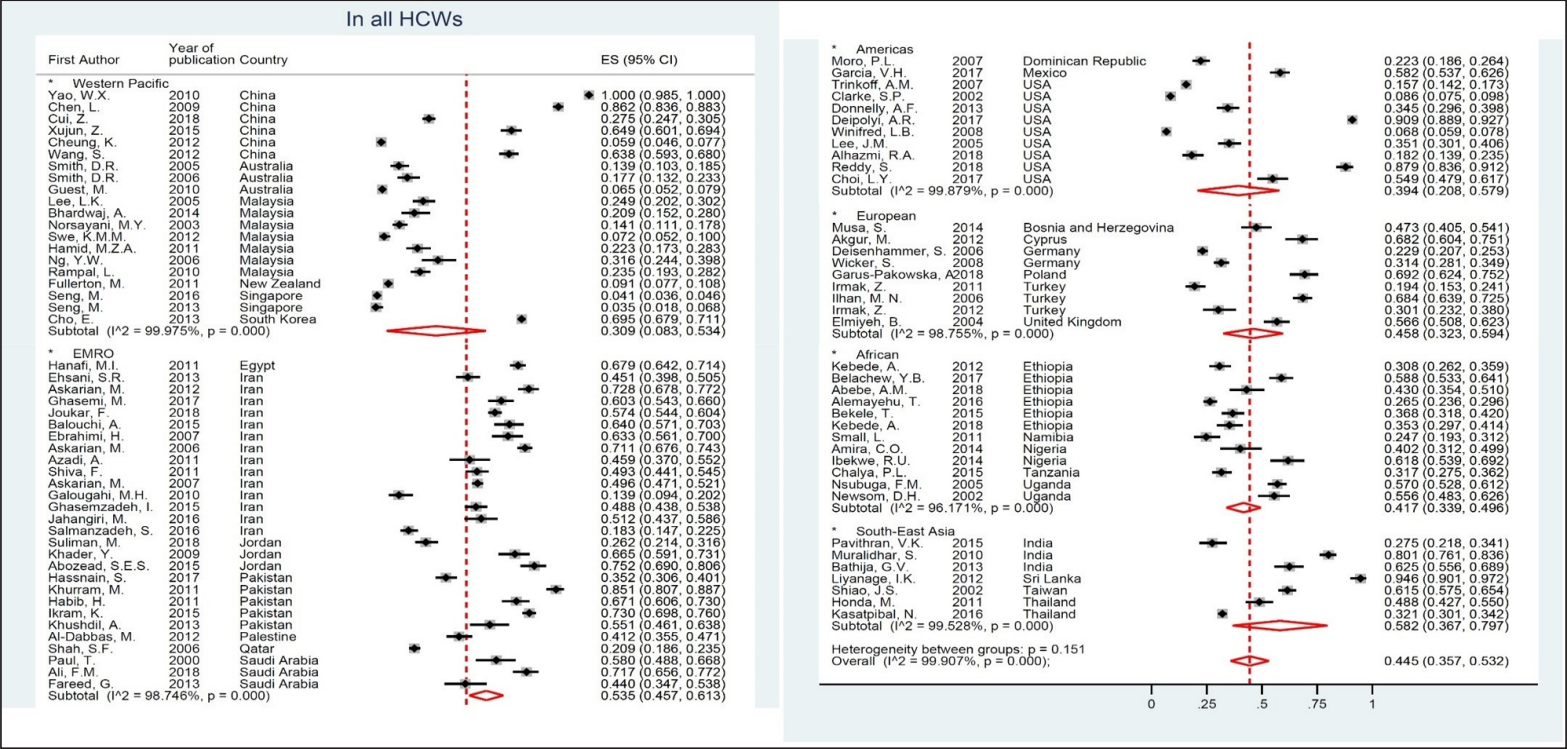
Global Prevalence of Needlestick injuries among health care workers based on WHO region.

**Figure 3:**
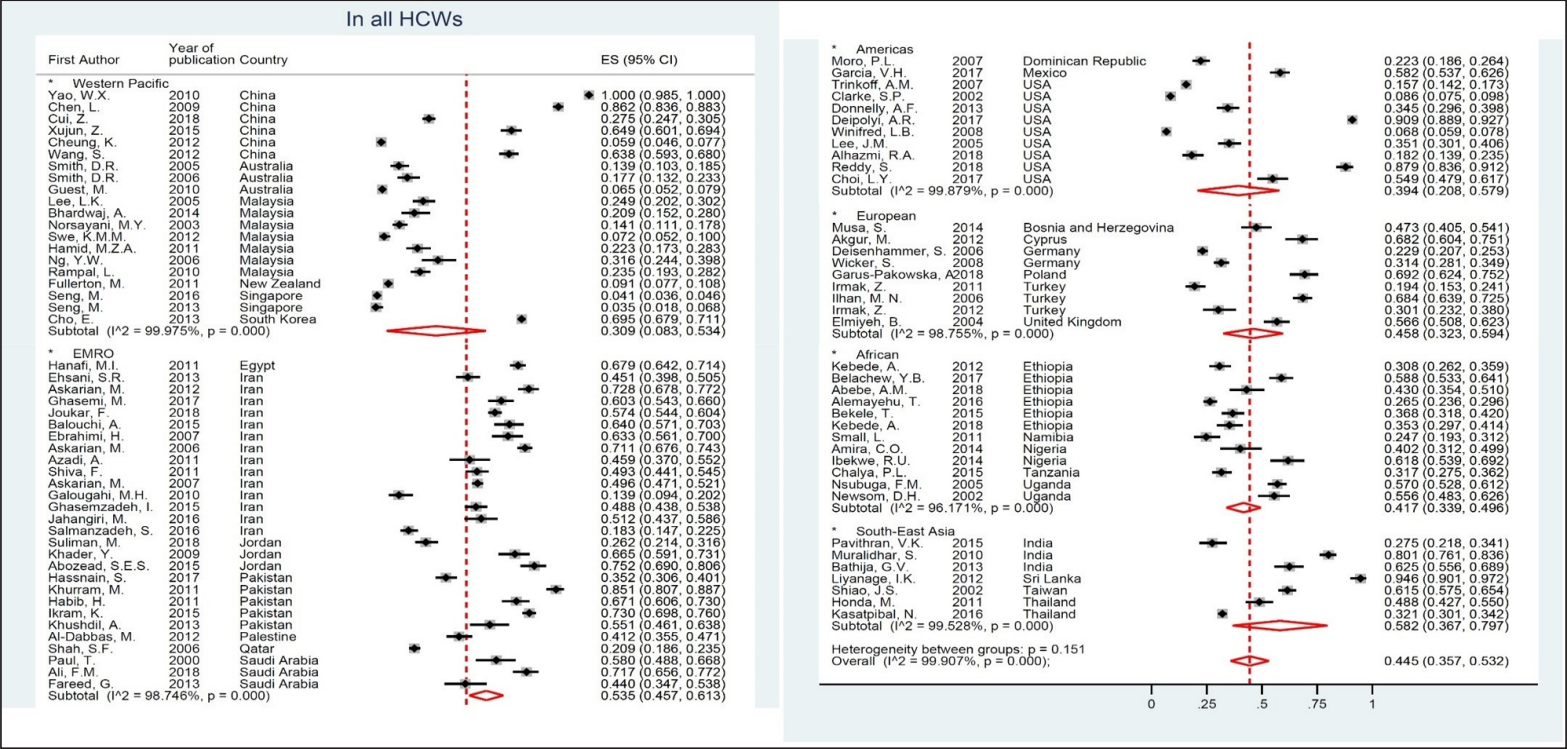
Global Prevalence of Needle stick injuries among health care workers based on Countries.

Prevalence of needle stick injuries (NSIs) (occurrence of at least 1 NSI within previous 12 months) was assessed in 87 studies conducted in 31 countries. Prevalence of NSIs in all HCWs was reported to be between 3.5 and 100%. Based on the results of the random effects method, the global prevalence of NSIs in all the 50,916 HCWs studied was 44.5% (95% CI: 35.7, 53.2; I^2^ = 99.9%). Sub-group analysis based on WHO regions showed that the pooled prevalence of NSIs in nurses was the lowest in Americans and the Western Pacific regions, and was the highest in the EMRO. The pooled prevalence in the EMRO was 1.9 times higher than that in America (52.0 vs. 26.7) and this difference was significant. The pooled prevalence in South-East Asia (58.2%) and the EMRO (53.5%) were higher than that in the Western Pacific (30.9%), America (39.4%), and the African (41.7%) regions (Figure 2). Among the 31 countries, the pooled prevalence in Singapore (based-on two studies), New Zealand (based-on one study), and Australia (based-on three studies) was lower than 15% and higher than 70% in Sri-Lanka (based-on one study) (Figure 3). Of the 31 studies, 18 studies reported NSI distribution by sex. Of the 2428 male and the 6009 female HCWs, 890 and 2357 NSIs were respectively reported globally. The pooled prevalence was slightly higher in women than in men (44.1% vs 39.8%), although this difference was not significant. Statistical difference was also observed between the WHO regions (Supplementary Figure 1).

Sub-group analysis based-on two time period, before and after 2010, did not show a significant difference in the prevalence of NSIs in the world, although in America region pooled prevalence in after 2010 was significantly 3.4 time more than before 2010. So, trend of NSIs prevalence only was significant for America region (P-value = 0.016) (Supplementary Table 4).

### Global prevalence of NSIs among HCWs based on type of job

#### Nurses

In this review, NSI data were extracted from eight groups of HCWs. NSIs were reported in 9739 of 28,197 nurses, the prevalence from 50 studies was between 2.6% and 100%, and the pooled prevalence was 42.8% (95% CI: 35.5, 50.1; I^2^ = 99.7%). Sub-group analysis based-on WHO regions showed that the pooled prevalence of NSIs in nurses was the lowest in the Western Pacific and America regions, and was the highest in South-East Asia, followed by the EMRO. The difference between the American and EMRO was significant and the pooled prevalence for the EMRO was 1.9 times higher than that in America (52.0 vs. 26.7) (Supplementary Figure 2).

#### Physicians

NSIs were reported in 1578 of 3602 physicians, the prevalence from 22 studies was between 10.5% and 86.2%, and the pooled prevalence was 46.4% (95% CI: 34.1, 58.8; I^2^ = 98.8%). Sub-group analysis based-on WHO regions did not show a significant difference in the pooled prevalence of NSIs in physicians (Supplementary Figure 3).

#### Nursing students

NSIs were reported in 1154 of 3197 nursing students, the prevalence from 11 studies was between 5.9% and 100%, and the pooled prevalence was 45.3% (95% CI: 11.2, 79.3; I^2^ = 99.9%). NSIs were reported in 1163 out of 3755 medical students, the prevalence in 12 studies was between 3.5% and 94.6%, and the pooled prevalence was 40.8% (95% CI: 23.1, 58.6; I^2^ = 99.6%).

#### Dentists

NSIs were reported in 889 of 1368 dentists, the prevalence from 4 studies was between 27.5% and 69.2%, and the pooled prevalence was 59.1% (95% CI: 38.8, 79.4; I^2^ = 98.2%). NSIs were reported in 266 out of 367 dentist students and the prevalence from 2 studies was between 71.7% and 73.7%. NSIs were reported in 221 out of 3822 HIV cases, the prevalence from 4 studies was between 1.0% and 58.6%, and the pooled prevalence was 11.5% (95% CI: 5.6, 17.3; I^2^ = 98.0%).

### Global prevalence of NSIs By socio-economic development (SDI)

Of all studies, 26 studies conducted in high socio-economic development (SDI), 39 study in high middle SDI, 13 and 9 studies in lower middle and low SDI, respectively. Sub-group analysis based-on socio-economic development (SDI) showed pooled prevalence of NSIs for all HCWs in high SDI was lower than other SDI groups and this difference was significant with lower middle SDI (37.6% vs. 61.0%). Also, in nurses and physician, the prevalence in high SDI were 2.2 and 1.9 time significantly lower than lower middle SDI (Table 1).

**Table 1:** Global Prevalence of Needle sticks injuries among health care workers based on SDI status and type of profession.

	All HCWs			In nurses			In physician			In medical students*		

	N	Pooled ES, % (95% CI)	I^2^, %	N	Pooled ES, % (95% CI)	I^2^, %	N	Pooled ES, % (95% CI)	I^2^, %	N	Pooled ES, % (95% CI)	I^2^, %

High SDI	26	37.6 (28.0, 47.3)	99.8	12	28.1 (16.5, 39.6)	99.8	6	39.6 (25.0, 54.2)	97.7	7	34.2 (16.0, 52.3)	99.4
High middle SDI	39	44.2 (30.6, 57.8)	99.9	26	45.0 (36.4, 53.5)	98.9	10	40.7 (21.0, 60.3)	98.3	10	34.7 (0.0, 71.0)	99.9
Low middle SDI	13	61.0 (50.0, 72.1)	98.5	6	62.9 (39.2, 86.7)	99.2	5	74.5 (63.9, 85.0)	90.8	5	70.6 (48.6, 94.7)	98.5
Low SDI	9	41.6 (35.8, 50.4)	96.5	6	43.1 (32.5, 53.6)	95.0	1	10.5 (5.6, 18.7)	NA	0	–	–
Global	87	44.5 (35.7, 53.2)	99.9	50	42.9 (35.0, 50.7)	99.7	22	46.4 (34.1, 58.8)	98.8	22	42.7 (20.7, 64.7)	99.9

* Total Students; SDI: Socio-economic development; N: Number of included studies; HCW: Health care worker; ES: Effect size; CI: Confidence interval.

### Meta-regression finding

The results of univariate meta-regression analyses, showed publication year of study, gender of participants (male-to-female ratio) and WHO regions variable not significantly contributed to heterogeneity of NSIs prevalence of HCWs in the world (P > 0.05); but, socio-economic development (SDI) showed a significant heterogeneity (P = 0.010), that explained 6.7% of between-study variation. Although, publication year of study did not explain heterogeneity in the world, but was significant in America regions [Coef. = 3.5% (95% CI: 0.81, 6.2), Adj R-squared = 43.7%, P-Value = 0.016].

### Device related causes of NSIs

Among the included studies, 40 studies reported hypodermic needle causes for the prevalence of NSIs, of which 25 studies reported suture needle, 18 reported IV cannula, 7 reported lancet, and 4 reported scalpel.

In these studies, of the 7065 NSI cases, 3223 were attributed to hypodermic needle causes with a prevalence between 4.7% and 100%. Of the 5849 NSI cases, 1152 were related to suture needle and the corresponding prevalence was between 2.0% and 57.1%. Of the 3913 NSI cases, 1034 were related to IV cannula and the corresponding prevalence was between 2.6% and 83.6%. Of the 1297 NSI cases, 89 were related to lancet and the corresponding prevalence was between 2.3% and 12.9%. Of the 1112 NSIs cases, 131 were related to scalpel and the corresponding prevalence was between 7.0% and 18.8%.

Based on the results of random effect method, between five NSIs causes, pooled prevalence of hypodermic needle was significantly higher than other causes [55.1% (95% CI: 41.4, 68.9; I^2^ = 99.7%)]. After hypodermic needle, pooled prevalence of IV cannula causes [23.0% (95% CI: 11.2, 34.8; I^2^ = 99.2%)] was prevalent and then suture needle [19.6% (95% CI: 14.8, 24.4; I^2^ = 97.4%)] and scalpel [16.8% (95% CI: 7.7, 26.0; I^2^ = 91.8%)]. Between causes, lancet cause was lower pooled prevalence of NSIs [7.0% (95% CI: 3.7, 10.2; I^2^ = 80.5%)].

## Discussion

NSI is one of the major safety challenges in the health care system worldwide. To the best of our knowledge, this is the first systematic review and meta-analysis that specifically examines the global prevalence of NSIs among HCWs. A total of 87 studies performed on 50,916 people from 31 countries were included in the final stage of the analysis. The global prevalence of NSIs among HCWs was 44.5%. The results of this study indicate a high prevalence of NSIs among HCWs. No prior review studies have focused on this purpose. However, a review aimed at evaluating percutaneous injuries, referred to NSIs as the most important cause for contact injuries (35.3%), which, in contrary to the present study, shows a higher prevalence of needle stick injuries (44.5%) [20]. Such a high prevalence can be attributed to demographic characteristics (old age and educational level), history of training on needle stick management, and number of shifts per month [21]. Additionally, needle sticking is a stressful process, especially if a person is exposed to high-risk patients such as hepatitis C and HIV that subsequently affect the mental health status of the individual. In this regard, studies show that 42% to 60% of HCWs suffer from stress and depression due to NSIs [22,23]. The prevalence of NSIs in the EMRO region was 2 times higher than that of the American and the Western Pacific regions. The lowest prevalence was observed in the American region, which is consistent with previous studies that examined percutaneous injuries [20,24,25].

Although the exact cause for the various prevalence rates in regions is unknown, the lower prevalence in developed regions such as Europe and the United States compared to other regions of the world may be due to the following: the difference in methodology and the number of studies included from each region in the present study, different rules, different methods and the level of supervision on the measurement of prevalence of needle stick injury in different WHO regions, the differences in national and regional policies in preventing needle stick injury, and fewer available details on precise prevention programs and annual national surveillance systems in less developed regions such as the EMRO. However, in developed countries, lower prevalence of NSIs could be due to the existence of comprehensive hospital-level NSI prevention programs, the provision of training courses and the provision of accurate information related to the management of NSIs, incentive systems for reporting NSI cases in hospitals, categorizing NSIs as a priority, establishing a preventive perspective on NSIs among HCWs, introducing practical policies including the use of new equipment to reduce NSIs, banning of recapping of needles, and providing HCWs with support in the event of NSIs, including: tests required, post exposure prophylaxis (PEP), counseling, rehabilitation and compensation for the financial and psychological damage of the affected person, and creating a safe work environment [26,27,28].

Among HCWs, NSIs were more prevalent among dentists. Unlike annual American studies and previous review studies, the highest prevalence of NSIs occurred among nurses [11,29]. In the present study, the higher prevalence of NSIs among dentists could be due to the nature of dentistry and the higher contact with sharp objects causing injuries. Based on previous studies, higher prevalence of NSIs among nurses shows the direct activity associated with NSIs in them [30].

Unlike countries with a middle-to-low SDI, in countries with a better economic situation, the prevalence of NSIs was lower. The NSI-related economic burden is a very important factor while addressing and preventing it in developed countries. The lower prevalence of NSIs in developed countries can be attributed to the allocation of sufficient budget to create a safe environment, prevention programs, and appropriate prevention equipment [12,31]. Globally, the most common causes of NSIs among HCWs were hypodermic needle (55.1%), IV cannula (23%), and needle suture (19.6%). The higher prevalence of hypodermic needle -induced NSIs may be due to the fact that the riskiest procedures are performed using syringe. IV cannula and suture needles cause NSIs injury less frequently.

Limitations: 1. The information was not fully reported in many of the included studies, and the authors were contacted for obtaining information. 2. Studies were carried out in only 31 countries of the world. 3. Most of the studies were from countries with a middle to high economic level, which limits the interpretation of results, especially for low income countries. 4. All the studies were cross-sectional, and special methodology limitations of these studies should be considered when interpreting their results. 5. The data were collected in a self-reported manner in most studies that may have affected the NSIs prevalence rate. Despite the aforementioned limitations, this study has many strengths. A systematic review and meta-analysis approach was used in the present study. Moreover, we have showed the global prevalence of NSIs based on income status in regions.

## Conclusion

In conclusion the results indicate a high global prevalence of NSIs among HCWs. The high prevalence of NSIs, despite existing strategies, indicates the inadequacy of current management strategies or the lack of adequate adherence of available standard precautions to prevent NSIs. Revising existing programs to integrate diverse programs in developed countries, as well as to apply the basic principles of NSIs prevention in less developed countries that do not follow a systematic NSIs management program, as well as the provision and implementation of standard training programs to enhance knowledge, performance, and creating a positive attitude among HCWs is vital. The results of this study can be used as a basis for planning by health policymakers and healthcare workers. Paying attention to the following items can reduce the NSI rate:

– Applying standard precautions.
– Periodic training to the HCWs on NSIs prevention and correct recapping.
– Develop a long-term NSIs reporting system for better management.
– Creating an appropriate safety and organizational culture among HCWs to encourage them for report of NSIs cases to the management.
– Establish clear and uniform policies across all hospitals about management of NSIs.
– Hospital infection control committees should regularly monitor the implementation of standard precautions guidelines.
– Perform periodic verbal and practical tests on staff knowledge, attitude, and performance regarding standard precautions of NSIs.

## Additional File

The additional file for this article can be found as follows:

10.5334/aogh.2698.s1 Supplementary file. PRISMA checklist, Keyword used for search, Demographic characteristics of included studies, and prevalence of NSI among physicians and nurses.
