# Supplementary material for: Global Prevalence and Device Related Causes of Needle Stick Injuries among Health Care Workers: A Systematic Review and Meta-Analysis
Source: Ann Glob Health. 2020 Apr 6;86(1):35. doi: 10.5334/aogh.2698 (PMC7181946; doi:10.5334/aogh.2698)
Supplement: Supplementary file. — PRISMA checklist, Keyword used for search, Demographic characteristics of included studies, and prevalence of NSI among physicians and nurses. [file agh-86-1-2698-s1.pdf]

Supplementary table 1. PRISMA 2009 Checklist

Section/topic	#	Checklist item	Reported on page #
**TITLE**			
Title	1	Identify the report as a systematic review, meta-analysis, or both.	1
**ABSTRACT**			
Structured summary	2	Provide a structured summary including, as applicable: background; objectives; data sources; study eligibility criteria, participants, and interventions; study appraisal and synthesis methods; results; limitations; conclusions and implications of key findings; systematic review registration number.	3
**INTRODUCTION**			
Rationale	3	Describe the rationale for the review in the context of what is already known.	4
Objectives	4	Provide an explicit statement of questions being addressed with reference to participants, interventions, comparisons, outcomes, and study design (PICOS).	5
**METHODS**			
Protocol and registration	5	Indicate if a review protocol exists, if and where it can be accessed (e.g., Web address), and, if available, provide registration information including registration number.	6
Eligibility criteria	6	Specify study characteristics (e.g., PICOS, length of follow-up) and report characteristics (e.g., years considered, language, publication status) used as criteria for eligibility, giving rationale.	6
Information sources	7	Describe all information sources (e.g., databases with dates of coverage, contact with study authors to identify additional studies) in the search and date last searched.	6
Search	8	Present full electronic search strategy for at least one database, including any limits used, such that it could be repeated.	6
Study selection	9	State the process for selecting studies (i.e., screening, eligibility, included in systematic review, and, if applicable, included in the meta-analysis).	7
Data collection process	10	Describe method of data extraction from reports (e.g., piloted forms, independently, in duplicate) and any processes for obtaining and confirming data from investigators.	7

Data items	11	List and define all variables for which data were sought (e.g., PICOS, funding sources) and any assumptions and simplifications made.	7
Risk of bias in individual studies	12	Describe methods used for assessing risk of bias of individual studies (including specification of whether this was done at the study or outcome level), and how this information is to be used in any data synthesis.	8
Summary measures	13	State the principal summary measures (e.g., risk ratio, difference in means).	8
Synthesis of results	14	Describe the methods of handling data and combining results of studies, if done, including measures of consistency (e.g., I^2) for each meta-analysis.	8

Section/topic	#	Checklist item	Reported on page #
Risk of bias across studies	15	Specify any assessment of risk of bias that may affect the cumulative evidence (e.g., publication bias, selective reporting within studies).	-
Additional analyses	16	Describe methods of additional analyses (e.g., sensitivity or subgroup analyses, meta-regression), if done, indicating which were pre-specified.	
**RESULTS**			
Study selection	17	Give numbers of studies screened, assessed for eligibility, and included in the review, with reasons for exclusions at each stage, ideally with a flow diagram.	9
Study characteristics	18	For each study, present characteristics for which data were extracted (e.g., study size, PICOS, follow-up period) and provide the citations.	9
Risk of bias within studies	19	Present data on risk of bias of each study and, if available, any outcome level assessment (see item 12).	10
Results of individual studies	20	For all outcomes considered (benefits or harms), present, for each study: (a) simple summary data for each intervention group (b) effect estimates and confidence intervals, ideally with a forest plot.	11-13
Synthesis of results	21	Present results of each meta-analysis done, including confidence intervals and measures of consistency.	11-13
Risk of bias across studies	22	Present results of any assessment of risk of bias across studies (see Item 15).	11-13
Additional analysis	23	Give results of additional analyses, if done (e.g., sensitivity or subgroup analyses, meta-regression [see Item 16]).	11-13
**DISCUSSION**			
Summary of evidence	24	Summarize the main findings including the strength of evidence for each main outcome; consider their relevance to key groups (e.g., healthcare providers, users, and policy makers).	14-15

Limitations	25	Discuss limitations at study and outcome level (e.g., risk of bias), and at review-level (e.g., incomplete retrieval of identified research, reporting bias).	16
Conclusions	26	Provide a general interpretation of the results in the context of other evidence, and implications for future research.	16
**FUNDING**			
Funding	27	Describe sources of funding for the systematic review and other support (e.g., supply of data); role of funders for the systematic review.	17

From: Moher D, Liberati A, Tetzlaff J, Altman DG, The PRISMA Group (2009). Preferred Reporting Items for Systematic Reviews and Meta-Analyses: The PRISMA Statement. PLoS Med 6(7): e1000097. doi:10.1371/journal.pmed1000097

For more information, visit: **www.prisma-statement.org**.

Supplementary Table 2. Keyword used for search

Occurrence OR "Prevalence" OR "Incidence" OR "Survey" OR "frequency" OR "rates or statistics" OR "performance indicators" OR "surveillance"	(Injury AND Needlestick) OR "Needlestick Injury" OR Needlestick* OR "Needle-Stick*" OR "Needle Stick*" OR (Injuri* AND Needlestick) OR "Needle-Stick Injuri*" OR "Needle Stick Injuries" OR "Needle-Stick Injur*) OR "Sharps Injuri*" OR "Sharp* Injury" OR sharp OR needle	dentists OR nurses OR Physician OR health workers OR health professionals OR (Personnel AND Health) OR "Health Care Provider*" OR (Provider* AND Health Care) OR "Healthcare Provider*" OR (Healthcare Worker*) OR "Registered Nurse"

Supplementary Table 3. Demographic characteristics of included studies

ID	First Author	Year	Country	Region	SDI status	sampling method	Target population	Type of Study setting	Participants	Risk of bias
1	Abebe, A. M.[1]	2018	Ethiopia	African	Low SDI	Simple Random	Nurses	Single-center	151	Low
2	Abozead, S. E. S. [2]	2015	Jordan	EMRO	High middle SDI	Census	Nurses	Multi-center	210	Low
3	Akgur, M. [3]	2012	Cyprus	European	High SDI	Census	Nurses	Single-center	151	Low
4	Al-Dabbas, M. [4]	2012	Palestine	EMRO	Low Middle SDI	Census	Medical Students	Multi-center	272	Low
5	Alemayehu, T. [5]	2016	Ethiopia	African	Low SDI	Simple Random	Nurse, Physician, Aids	Multi-center	820	Low
6	Alhazmi, R. A. [6]	2018	USA	Americas	High SDI	Simple Random	Other	Multi-center	247	Low
7	Ali, F. M. [7]	2018	Saudi Arabia	EMRO	High SDI	Simple Random	Dental students	Single-center	230	Low
8	Amira, C. O. [8]	2014	Nigeria	African	Low middle SDI	Census	Nurse, Physician, Aids	Multi-center	102	Low
9	Askarian, M. [9]	2007	Iran	EMRO	High middle SDI	Simple Random	nurse, Aids	Multi-center	1555	Low
10	Askarian, M. [10]	2012	Iran	EMRO	High middle SDI	Convenience	Nursing, dental students	Multi-center	345	Low
11	Askarian, M. [11]	2006	Iran	EMRO	High middle SDI	Convenience	Nursing, medical, dental students	Single-center	688	Low
12	Azadi, A. [12]	2011	Iran	EMRO	High	Simple	Nurses	Multi-center	111	Low

					middle SDI	Random				
13	Balouchi, A. [13]	2015	Iran	EMRO	High middle SDI	Simple Random	Nurses	Single-center	200	Low
14	Bathija, G. V. [14]	2013	India	South-East Asia	Low middle SDI	Convenience	Nursing, medical students	Single-center	200	Low
15	Bekele, T.[15]	2015	Ethiopia	African	Low SDI	Convenience	HCWs	Multi-center	340	Low
16	Belachew, Y.B. [16]	2017	Ethiopia	African	Low SDI	Census	Nurses	Multi-center	318	Low
17	Bhardwaj, A. [17]	2014	Malaysia	Western Pacific	High middle SDI	Census	Nurse, Physician	Single-center	153	Low
18	Chalya, P. L. [18]	2015	Tanzania	African	Low SDI	Census	nurse, Physician, Aids , others	Single-center	436	Low
19	Chen, L [19]	2009	China	Western Pacific	High middle SDI	Census	Nurse, Physician, others	Multi-center	831	Low
20	Cheung, K. [20]	2012	China	Western Pacific	High middle SDI	Convenience	Nursing Students	Multi-center	877	Low
21	Cho, E. [21]	2013	South Korea	Western Pacific	High SDI	Stratified Random	Nurses	Multi-center	3079	Low
22	Choi, L. Y. [22]	2017	USA	Americas	High SDI	Census	Nurse, Physician, Medical Students	Single-center	195	Low
23	Clarke, S. P. [23]	2002	USA	Americas	High SDI	Census	Nurses	Multi-Centre	2287	Low
24	Cui, Z. [24]	2018	China	Western Pacific	High middle SDI	Census	Nurse, Physician	Single-center	901	Low
25	Deipolyi, A. R. [25]	2017	USA	Americas	High SDI	Census	Other	Multi-Centre	895	Low
26	Deisenhammer,	2006	Germany	European	High SDI	Census	Medical Students	Multi-Centre	1317	Low

	S. [26]									
27	Donnelly, A. F. [27]	2013	USA	Americas	High SDI	Census	Physicians	Multi-center	336	Low
28	Ebrahimi, H. [28]	2007	Iran	EMRO	High middle SDI	Census	Nurses	Single-center	180	Low
29	Ehsani, S. R. [29]	2013	Iran	EMRO	High middle SDI	Census	Nurses	Multi-center	328	Low
30	Elmiyeh, B [30]	2004	United Kingdom	European	High SDI	Census	HCWs	Multi-center	279	Low
31	Fareed, G. [31]	2013	Saudi Arabia	EMRO	High SDI	Census	Nursing Students	Single-center	100	Low
32	Fullerton, M. [32]	2011	New Zealand	Western Pacific	High SDI	Census	Nurse, Physician, others	Multi-center	1346	Low
33	Galougahi, M. H. [33]	2010	Iran	EMRO	High middle SDI	Consecutive	Nurses	Single-center	158	Low
34	Garcia, V. H. [34]	2017	Mexico	Americas	High middle SDI	Census	HCWs	Multi-center	467	Low
35	Garus-Pakowska, A. [35]	2018	Poland	European	High SDI	UK	Dentists	Multi-center	198	Low
36	Ghasemi, M. [36]	2017	Iran	EMRO	High middle SDI	Stratified Random	Nurses	Single-center	267	Low
37	Ghasemzadeh, I. [37]	2015	Iran	EMRO	High middle SDI	Census	Nurse, Medical Students	Single-center	377	Low
38	Guest, M. [38]	2010	Australia	Western Pacific	High SDI	Stratified Random	Nurses	Single-center	1301	Low
39	Habib, H. [39]	2011	Pakistan	EMRO	Low middle	Simple	Nurses	Single-center	216	Low

					SDI	Random				
40	Hamid, M. Z. A. [40]	2011	Malaysia	Western Pacific	High middle SDI	Universal sampling	Nurse, Physician	Multi-center	215	Low
41	Hanafi, M. I. [41]	2011	Egypt	EMRO	Low middle SDI	Census	Nurse, Physician, others	Multi-center	645	Low
42	Hassnain, S. [42]	2017	Pakistan	EMRO	Low middle SDI	Simple Random	Nurses	Multi-center	386	Low
43	Honda, M. [43]	2011	Thailand	South-East Asia	High middle SDI	Stratified Random	Nurses	Multi-center	250	Low
44	Ibekwe, R. U. [44]	2014	Nigeria	African	Low middle SDI	Census	Physicians	Multi-center	152	Low
45	Ikram, K. [45]	2015	Pakistan	EMRO	Low middle SDI	Census	Dentists	Multi-center	800	Low
46	Ilhan, M. N. [46]	2006	Turkey	European	High middle SDI	Census	Nurses	Multi-center	449	Low
47	Irmak, Z. [47]	2012	Turkey	European	High middle SDI	Census	Nurses	Multi-center	143	Low
48	Irmak, Z. [48]	2011	Turkey	European	High middle SDI	Census	Nursing Students	Single-center	310	Low
49	Jahangiri, M. [49]	2016	Iran	EMRO	High middle SDI	Simple Random	Nurses	Multi-center	168	Low
50	Joukar, F. [50]	2018	Iran	EMRO	High middle SDI	Consecutive	Nurses	Multi-center	1010	Low
51	Kasatpibal, N. [51]	2016	Thailand	South-East Asia	High middle SDI	Stratified Random	Nurses	Multi-center	2031	Low
52	Kebede, A. [52]	2012	Ethiopia	African	Low SDI	Simple Random	nurse, Physician, Aids	Multi-center	344	Low

53	Kebede, A. [53]	2018	Ethiopia	African	Low SDI	Simple Random	Nurses	Multi-center	252	Low
54	Khader, Y. [54]	2009	Jordan	EMRO	High middle SDI	Simple Random	Dentists	Multi-center	170	Low
55	Khurram, M. [55]	2011	Pakistan	EMRO	Low middle SDI	Census	Physicians	Single-center	309	Low
56	Khushdil, A. [56]	2013	Pakistan	EMRO	Low middle SDI	Census	Nurses	Multi-center	118	Low
57	Lee, J. M. [57]	2005	USA	Americas	High SDI	Census	Nurses	Multi-center	313	Low
58	Lee, L. K. [58]	2005	Malaysia	Western Pacific	High middle SDI	Simple Random	Nurse, Physician, Medical Students	Multi-center	285	Low
59	Liyanage IK [59]	2012	Sri Lanka	South-East Asia	Low middle SDI	Census	Medical Students	Single-center	168	Low
60	Moro, P. L. [60]	2007	Dominican Republic	Americas	High middle SDI	Simple Random	Nurse, Physician, others	Multi-center	440	Low
61	Muralidhar, S. [61]	2010	India	South-East Asia	Low middle SDI	Census	Nurse, Physician, Nurse, Medical students	Single-center	428	Low
62	Musa, S. [62]	2014	Bosnia and Herzegovina	European	High middle SDI	Simple Random	nurse, Physician, Aids	Single-center	203	Low
63	Newsom, D. H. [63]	2002	Uganda	African	Low SDI	Consecutive	nurse, Physician, medical students, other	Single-center	180	Low
64	Ng, Y.W. [64]	2007	Malaysia	Western Pacific	High middle SDI	Convenience	Nurse, Physician, others	Multi-center	136	Low
65	Norsayani, M. Y. [65]	2003	Malaysia	Western Pacific	High middle SDI	Universal sampling	Medical Students	Multi-center	417	Low

66	Nsubuga, F. M. [66]	2005	Uganda	African	Low SDI	Census	nurse, Aids, others	Single-center	526	Low
67	Paul, T. [67]	2000	Saudi Arabia	EMRO	High SDI	Census	Dentists	Multi-center	112	Low
68	Pavithran, V. K. [68]	2015	India	South-East Asia	Low middle SDI	Convenience	Dentists	Multi-center	200	Low
69	Rampal, L. [69]	2010	Malaysia	Western Pacific	High middle SDI	Simple Random	Nurse, Physician, others	Single-center	345	Low
70	Reddy, S. [70]	2018	USA	Americas	High SDI	Consecutive	Other	Multi-center	282	Low
71	Salmanzadeh, S. [71]	2016	Iran	EMRO	High middle SDI	Simple Random	nurse, Physician, Aids, others	Single-center	377	Low
72	Seng, M. [72]	2013	Singapore	Western Pacific	High SDI	Simple Random	Medical Students	Single-center	228	Low
73	Seng, M. [73]	2016	Singapore	Western Pacific	High SDI	Simple Random	nurse, Physician, Aids, others	Multi-center	5957	Low
74	Shah, S. F. [74]	2006	Qatar	EMRO	High SDI	Simple Random	Nurse, Physician, others	Single-center	1022	Low
75	Shiao, J. S. [75]	2002	Taiwan	South-East Asia	High SDI	Simple Random	Nursing Students	Multi-center	572	Low
76	Shiva, F. [76]	2011	Iran	EMRO	High middle SDI	Simple Random	Nurse, Physician	Multi-center	355	Low
77	Small, L. [77]	2011	Namibia	African	High middle SDI	Convenience	Nursing Students	Single-center	198	Low
78	Smith, D. R. [78]	2006	Australia	Western Pacific	High SDI	Convenience	Nurses	Multi-center	220	Low
79	Smith, D. R. [79]	2005	Australia	Western Pacific	High SDI	Convenience	Nursing Students	Single-center	274	Low

80	Suliman, M. [80]	2018	Jordan	EMRO	High middle SDI	Convenience	Nursing Students	Multi-center	279	Low
81	Swe, K. M. M. [81]	2012	Malaysia	Western Pacific	High middle SDI	Census	Medical Students	Single-center	456	Low
82	Trinkoff, A. M. [82]	2007	USA	Americas	High SDI	Convenience	Nurses	Multi-center	2237	Low
83	Wang, S. [83]	2012	China	Western Pacific	High middle SDI	Convenience	Nurses	Single-center	458	Low
84	Wicker, S. [84]	2008	Germany	European	High SDI	Convenience	Nurse, Physician, others	Single-center	720	Low
85	Winifred, LB. [85]	2008	USA	Americas	High SDI	Simple Random		Multi-center	2664	Low
86	Xujun, Z. [86]	2015	China	Western Pacific	High middle SDI	Convenience	Nurses	Single-center	402	Low
87	Yao, W. X. [87]	2010	China	Western Pacific	High middle SDI	Convenience	Nursing Students	Multi-center	246	Low

UK: unknown, M: month

Supplementary Figure 1. Global Prevalence of NSIs injuries among health care workers based on gender

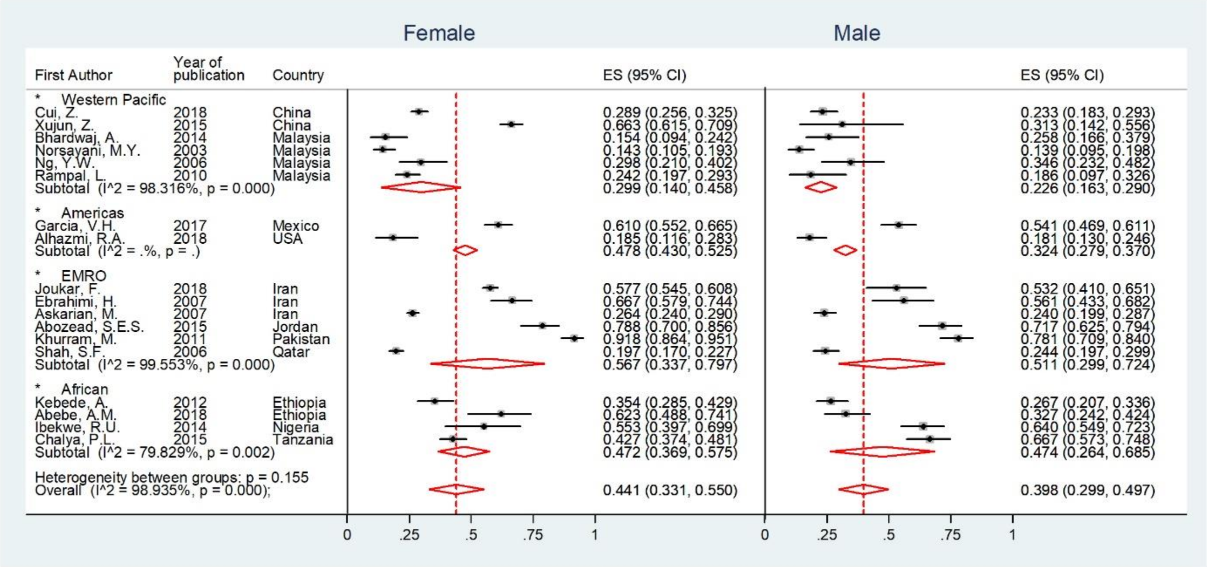

Supplementary Table 4; Global Prevalence of Needle sticks injuries in two time period and prevalence trend by WHO region

	**2000-2010**			**2010-2018**			**Prevalence trend**		
	**N**	**Pooled ES, % (95% CI)**	**I2, %**	**N**	**Pooled ES, % (95% CI)**	**I2, %**	**Coef.*, %**	**95% CI for Coef.**	**P-value**
**America**	5	17.0 (11.5, 22.6)	98.3	6	57.5 (32.8, 82.2)	99.6	3.5	0.81, 6.2	0.016
**Africa**	5	45.9 (31.1, 60.7)	97.0	7	38.7 (30.2, 47.3)	94.7	-0.90	-2.7, 0.87	0.283
**EMRO**	7	49.0 (31.5, 66.4)	99.2	21	55.0 (45.7, 61.3)	98.2	-0.22	-1.9, 1.5	0.794
**Europe**	4	44.7 (24.0, 65.5)	99.4	5	46.8 (25.3, 68.2)	98.3	0.90	-3.1, 4.9	0.612
**South-East Asia**	2	71.3 (68.6, 74.1)	NA	5	53.1 (23.6, 82.6)	99.6	-2.0	-7.6, 3.6	0.392
**Western Pacific**	9	35.4 (0.0, 72.0)	99.9	11	27.1 (12.5, 41.8)	99.8	0.47	-3.2, 4.1	0.791
**Global**	**29**	**40.8 (23.4, 58.3)**	**99.9**	**58**	**46.3 (37.9, 54.7)**	**99.8**	**0.71**	**-0.43, 1.8**	**0.219**

* Coefficient of meta-regression analysis; ES: Effect size; CI: Confidence interval

Supplementary Figure 2. Global Prevalence of Needlestick injuries (NSIs) among Nurses

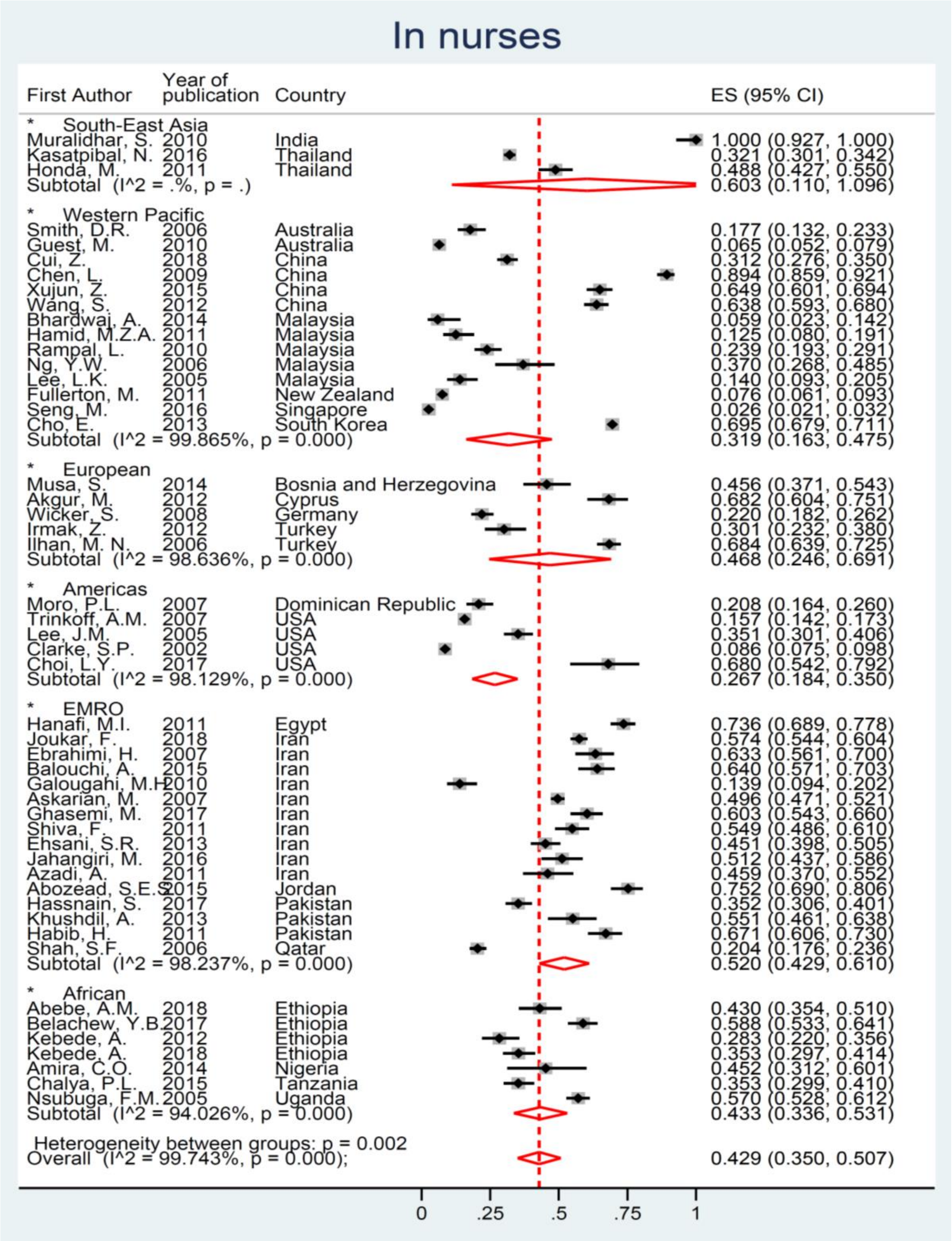

Supplementary Figure 3. Global Prevalence of Needlestick injuries (NSIs) among physicians

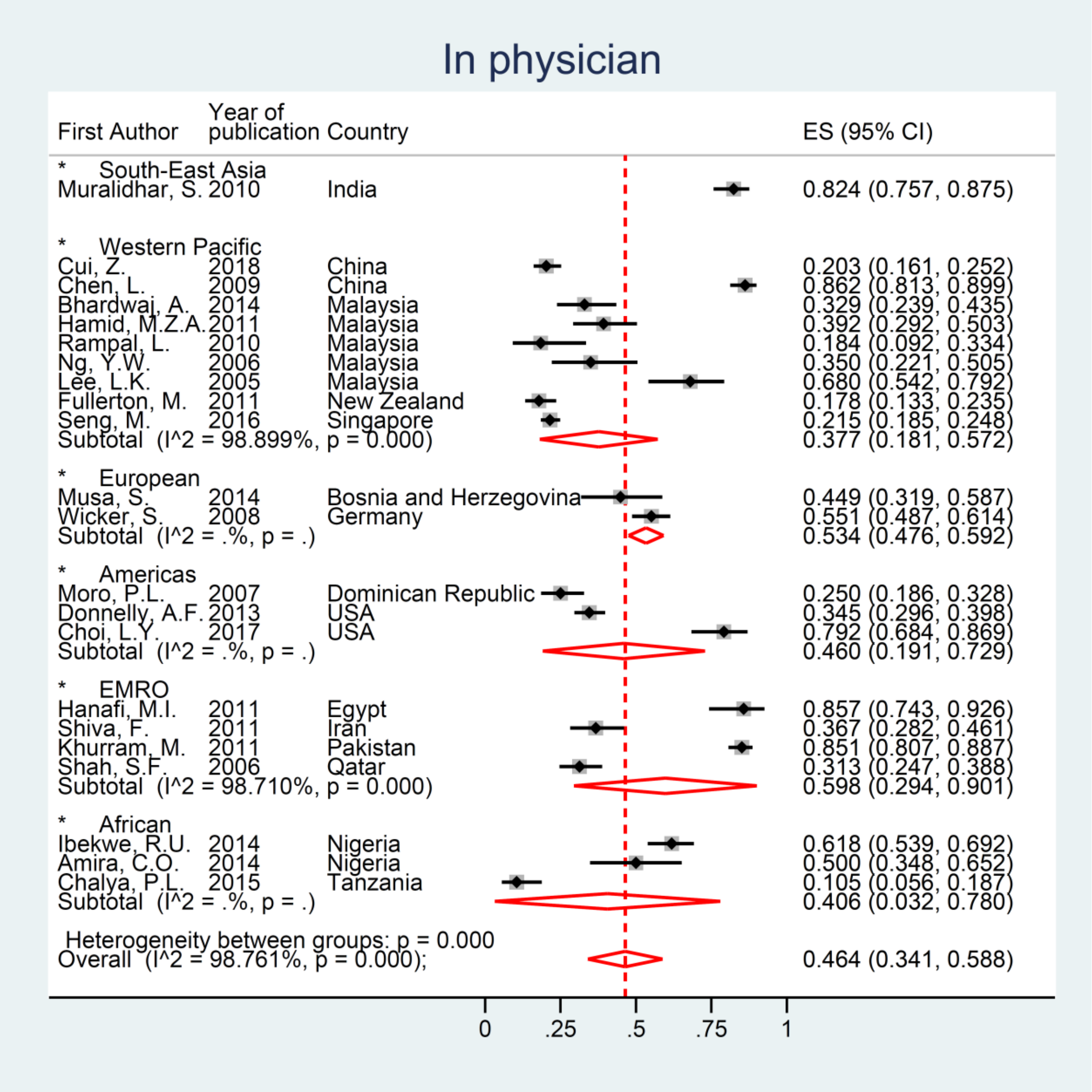

References

.1 Abebe AM, Kassaw MW, Shewangashaw NE. Prevalence of needle-stick and sharp object injuries and its associated factors among staff nurses in Dessie referral hospital Amhara region, Ethiopia, 2018. BMC research notes **2018**; 11(1): 840.

.2 Abozead SES, Abuhasheesh M, Nawafleh H, Kawafha MM, Al-Tarawneh O. Knowledge and practices of jordanian nurses on needlestick injuries: An evaluative study. Infect Dis Clin Pract **2015**; 23(1): 21-5.

.3 Akgur M, Dal U. The prevalence of needle stick and sharps injuries in nurses in North Cyprus. Pak J Med Sci **2012**; 28(3): 437-40.

.4 Al-Dabbas M, Abu-Rmeileh NME. Needlestick injury among interns and medical students in the Occupied Palestinian Territory. East Mediterr Health J **2012**; 18(7): 700-6.

.5 Alemayehu T, Worku A, Assefa N. Sharp Injury and Exposure to Blood and Body Fluids among Health Care Workers in Health Care Centers of Eastern Ethiopia. The international journal of occupational and environmental medicine **2016**; 7(3): 172-80.

.6 Alhazmi RA, Parker RD, Wen S. Needlestick Injuries Among Emergency Medical Services Providers in Urban and Rural Areas. Journal of Community Health **2018**; 43(3): 518-23.

.7 Ali FM, Al-Iryani GM, Mdabesh HY, et al. Incidence of needlestick injuries in oral surgery clinic among dental students: A retrospective study. World J Dent **2018**; 9(1): 29-33.

.8 Amira CO, Awobusuyi JO. Needle-stick injury among health care workers in hemodialysis units in Nigeria: a multi-center study. The international journal of occupational and environmental medicine **2014**; 5(1): 1-8.

.9 Askarian M, Shaghaghian S, McLaws ML. Needlestick injuries among nurses of Fars province, Iran. Annals of epidemiology **2007**; 17(12): 988-92.

.10 Askarian M, Malekmakan L, Memish ZA, Assadian O. Prevalence of needle stick injuries among dental, nursing and midwifery students in Shiraz, Iran. GMS Krankenhaushygiene interdisziplinar **2012**; 7(1): Doc05.

.11 Askarian M, Malekmakan L. The prevalence of needle stick injuries in medical, dental, nursing and midwifery students at the University Teaching Hospitals of Shiraz, Iran. Indian J Med Sci **2006**; 60(6): 227-32.

.12 Azadi A, Anoosheh M, Delpisheh A. Frequency and barriers of underreported needlestick injuries amongst Iranian nurses, a questionnaire survey. Journal of clinical nursing **2011** ; .93-488 :(4-3)20

.13 Balouchi A, Shahdadi H, Ahmadidarrehsima S, Rafiemanesh H. The Frequency, Causes and Prevention of Needlestick Injuries in Nurses of Kerman: A Cross-Sectional Study. J Clin Diagn Res **2015**; 9(12): Dc13-5.

.14 Bathija GV, Bant DD, Itagimath SR. A study on prevalence of needle stick injuries among junior doctors and nursing students in Kims, Hubli. Indian J Public Health Res Dev **2013**; 4(2): 84-8.

.15 Bekele T, Gebremariam A, Kaso M, Ahmed K. Attitude, reporting behavour and management practice of occupational needle stick and sharps injuries among hospital healthcare workers in Bale zone, Southeast Ethiopia: a cross-sectional study. Journal of occupational medicine and toxicology (London, England) **2015**; 10: 42.

.16 Belachew YB, Lema TB ,Germossa GN, Adinew YM. Blood/Body Fluid Exposure and Needle Stick/Sharp Injury among Nurses Working in Public Hospitals; Southwest Ethiopia. Frontiers in public health **2017**; 5: 299.

.17 Bhardwaj A, Sivapathasundaram N, Yusof M, Minghat A, Swe K, Sinha N. The Prevalence of Accidental Needle Stick Injury and their Reporting among Healthcare Workers in Orthopaedic Wards in General Hospital Melaka, Malaysia. Malaysian orthopaedic journal **2014**; 8(2): 6-13.

.18 Chalya PL, Seni J, Mushi MF, et al. Needle-stick injuries and splash exposures among health-care workers at a tertiary care hospital in north-western Tanzania. Tanzan J Health Res **2015**; 17.(2)
.19 Chen L, Zhang M, Yan Y, et al. Sharp object injuries among health care workers in a Chinese province. AAOHN journal : official journal of the American Association of Occupational Health Nurses **2009**; 57(1): 13-6.
.20 Cheung K, Ching SS, Chang KK, Ho SC. Prevalence of and risk factors for needlestick and sharps injuries among nursing students in Hong Kong. American journal of infection control **2012**; 40(10): 997-1001.
.21 Cho E, Lee H, Choi M, Park SH, Yoo IY, Aiken LH. Factors associated with needlestick and sharp injuries among hospital nurses: a cross-sectional questionnaire survey. International journal of nursing studies **2013**; 50(8): 1025-32.
.22 Choi LY, Torres R, Syed S, et al. Sharps and Needlestick Injuries Among Medical Students, Surgical Residents, Faculty, and Operating Room Staff at a Single Academic Institution. Journal of surgical education **2017**.6-131 :(1)74 ;
.23 Clarke SP, Rockett JL, Sloane DM, Aiken LH. Organizational climate, staffing, and safety equipment as predictors of needlestick injuries and near-misses in hospital nurses. American journal of infection control **2002**; 30(4): 207-16.
.24 Cui Z, Zhu J, Zhang X, Wang B, Li X. Sharp injuries: a cross-sectional study among health care workers in a provincial teaching hospital in China. Environmental health and preventive medicine **2018**; 23(1): 2.
.25 Deipolyi AR, Prabhakar AM, Naidu S, Oklu R .Needlestick Injuries in Interventional Radiology Are Common and Underreported. Radiology **2017**; 285(3): 870-5.
.26 Deisenhammer S, Radon K, Nowak D, Reichert J. Needlestick injuries during medical training. J Hosp Infect **2006**; 63(3): 263-7.
.27 Donnelly AF, Chang YH, Nemeth-Ochoa SA. Sharps injuries and reporting practices of U.S. dermatologists. Dermatologic surgery : official publication for American Society for Dermatologic Surgery [et al] **2013**; 39(12): 1813-21.
.28 Ebrahimi H, Khosravi A. Needlestick Injuries among Nurses. Journal of research in health sciences **2007**; 7(2): 56-62.
.29 Ehsani SR, Mohammadnejad E, Hadizadeh MR, et al. Epidemiology of needle sticks and sharp injuries among nurses in an Iranian Teaching Hospital. Arch Clin Infect Dis **2013**.30-27 :(1)8 ;
.30 Elmiyeh B, Whitaker IS, James MJ, Chahal CA, Galea A, Alshafi K. Needle-stick injuries in the National Health Service: a culture of silence. Journal of the Royal Society of Medicine **2004**; 97(7): 326-7.
.31 Fareed G, Hussain A, Jameel A ,Akhtar M, Hussain A, Taj S. Knowledge, attitude and practices among nursing students on needle-stick injuries. Pak J Med Health Sci **2013**; 7.(2)
.32 Fullerton M, Gibbons V. Needlestick injuries in a healthcare setting in New Zealand. New Zealand Med J **2011**.9-33 :(1335)124 ;
.33 Galougahi MH. Evaluation of needle stick injuries among nurses of Khanevadeh Hospital in Tehran. Iranian journal of nursing and midwifery research **2010**; 15(4): 172-7.
.34 Garcia VH, Radon K. Preventive training among medical interns in Mexico city and its association with needlestick and sharp injuries – A cross sectional study. J Clin Diagn Res **2017**; 11(3): IC05-IC7.
.35 Garus-Pakowska A, Górajski M, Gaszyńska E. Occupational safety and hygiene of dentists from urban and rural areas in terms of sharp injuries: Wound structure, causes of injuries and barriers to reporting—cross-sectional study, Poland. Int J Environ Res Public Health **2018**; 15.(8)
.36 Ghasemi M, Khabazkhoob M, Hashemi H, Yekta A, Nabovati P. The incidence of needle stick and sharp injuries and their associations with visual function among hospital nurses. Journal of current ophthalmology **2017**; 29(3): 214-20.

.37 Ghasemzadeh I, Kazerooni M, Davoodian P, Hamedi Y, Sadeghi P. Sharp Injuries Among Medical Students. Glob J Health Sci **2015**; 7(5): 320-5.
.38 Guest M, Kable A, McLeod M. A survey of sharps including needlestick injuries in nurses in New South Wales, Australia. Healthc Infect **2010**; 15(3): 77-83.
.39 Habib H, Khan EA, Aziz A. Prevalence and factors associated with needle stick injuries among registered nurses in public sector tertiary care hospitals of Pakistan. Int J Collab Res Intern Med Public Health **2011**; 3(2): 124-30.
.40 Hamid MZA, Aziz NA, Lim WB, et al. Prevalence of Needle Stick Injuries and Compliance to Infection Control Guidelines Among Health Care Workers in a Teaching Hospital, Malaysia. Malays J Med Health Sci **2011**; 7(1): 3-7.
.41 Hanafi MI, Mohamed AM, Kassem MS, Shawki M. Needlestick injuries among health care workers of University of Alexandria hospitals. East Mediterr Health J **2011**; 17(1): 26-35.
.42 Hassnain S, Hassan Z, Amjad S, Zulqarnain M, Arshad K, Zain Z. Needle stick injuries among nurses of two tertiary care hospitals of Lahore: A KAP study. JPMA The Journal of the Pakistan Medical Association **2017**; 67(12): 1874-8.
.43 Honda M, Chompikul J, Rattanapan C, Wood G, Klungboonkrong S. Sharps injuries among nurses in a Thai regional hospital: prevalence and risk factors. The international journal of occupational and environmental medicine.23-215 :(4)2 ;2011
.44 Ibekwe RU, Adam VY. Injection safety practices among resident doctors in a tertiary health facility in Benin City. Nigerian journal of clinical practice **2014**; 17(4): 403-6.
.45 Ikram K, Siddiqui HK, Maqbool S, Altaf M, Khan S. Frequency of needle stick injury among dental students and dentists of Karachi. World J Dent **2015**; 6(4): 213-6.
.46 Ilhan MN, Durukan E, Aras E, Turkcuoglu S, Aygun R. Long working hours increase the risk of sharp and needlestick injury in nurses: the need for new policy implication. Journal of advanced nursing **2006**; 56(5): 563-8.
.47 Irmak Z. Needlestick and sharps injury among nurses at a state hospital in Turkey. Aus J Adv Nurs **2012**; 30(2): 48-55.
.48 Irmak Z, Baybuga MS. Needlestick and sharps injuries among Turkish nursing students: A cross-sectional study. Int J Nurs Pract **2011**; 17(2): 151-7.
.49 Jahangiri M, Rostamabadi A, Hoboubi N, Tadayon N, Soleimani A. Needle Stick Injuries and their Related Safety Measures among Nurses in a University Hospital, Shiraz, Iran. Safety and health at work **2016**; 7(1): 72-7.
.50 Joukar F, Mansour-Ghanaei F, Naghipour M, Asgharnezhad M. Needlestick Injuries among Healthcare Workers: Why They Do Not Report their Incidence? Iranian journal of nursing and midwifery research.7-382 :(5)23 ;2018
.51 Kasatpibal N, Whitney JD, Katechanok S, et al. Prevalence and risk factors of needlestick injuries, sharps injuries, and blood and body fluid exposures among operating room nurses in Thailand. American journal of infection control.90-85 :(1)44 ;2016
.52 Kebede G, Molla M, Sharma HR. Needle stick and sharps injuries among health care workers in Gondar city, Ethiopia. Saf Sci **2012**; 50(4): 1093-7.
.53 Kebede A, Gerensea H. Prevalence of needle stick injury and its associated factors among nurses working in public hospitals of Dessie town, Northeast Ethiopia, 2016. BMC research notes **2018**; 11(1): 413.
.54 Khader Y, Burgan S, Amarin Z. Self-reported needle-stick injuries among dentists in north Jordan. Eastern Mediterranean health journal = La revue de sante de la Mediterranee orientale = al-Majallah al-sihhiyah li-sharq al-mutawassit **2009**; 15(1): 185-9.
.55 Khurram M, Ijaz K, Bushra HT, Khan NY, Bushra H, Hussain W. Needlestick injuries: a survey of doctors working at Tertiary Care Hospitals of Rawalpindi. JPMA The Journal of the Pakistan Medical Association **2011**; 61(1): 63-5.
.56 Khushdil A, Farrukh H, Sabir MD, Awan T, Qureshi T. Needle stick injuries in nurses at a tertiary health care facility. J Postgrad Med Inst **2013**; 27(4): 38.6-1

.57 Lee JM, Botteman MF, Nicklasson L, Cobden D, Pashos CL. Needlestick injury in acute care nurses caring for patients with diabetes mellitus: a retrospective study. Current medical research and opinion **2005**; 21(5): 741-7.
.58 Lee LK, Noor Hassim I .Implication of the prevalence of needlestick injuries in a general hospital in Malaysia and its risk in clinical practice. Environ Health Prev Med **2005**; 10(1): 33-41.
.59 Liyanage IK, Caldera T, Rwma R, Liyange C, De Silva P, Karunathilake I. Sharps injuries among medical students in the Faculty of Medicine, Colombo, Sri Lanka. Int J Occup Med Environ Health **2012**; 25(3): 275-80.
.60 Moro PL, Moore A, Balcacer P, et al. Epidemiology of needlesticks and other sharps injuries and injection safety practices in the Dominican Republic. American journal of infection control **2007**; 35(8): 552-9.
.61 Muralidhar S, Singh PK, Jain RK, Malhotra M, Bala M. Needle stick injuries among health care workers in a tertiary care hospital of India. The Indian journal of medical research **2010**; 131: 405-10.
.62 Musa S, Peek-Asa C, Young T, Jovanovic N. Needle Stick Injuries, Sharp Injuries and other Occupational Exposures to Blood and Body Fluids among Health Care Workers in a general hospital in Sarajevo, Bosnia and Herzegovina. International journal of occupational safety and health **2014**; 4(1): 31-7.
.63 Newsom DH, Kiwanuka JP. Needle-stick injuries in an Ugandan teaching hospital. Annals of tropical medicine and parasitology **2002**; 96(5): 517-22.
.64 Ng YW, Noor Hassim I. Needlestick injury among medical personnel in accident and emergency department of two teaching hospitals. Med J Malays **2007**; 62(1): 9-12.
.65 Norsayani MY, Hassim IN. Study on incidence of needle stick injury and factors associated with this problem among medical students. J Occup Health **2003**; 45(3): 172-8.
.66 Nsubuga FM, Jaakkola MS. Needle stick injuries among nurses in sub-Saharan Africa. Tropical medicine & international health : TM & IH **2005**; 10(8): 773-81.
.67 Paul T, Omar R, Maktabi A. Self-reported needlestick injuries in dental health care workers at Armed Forces Hospital Riyadh, Saudi Arabia. Mil Med **2000**; 165(3): 208-10.
.68 Pavithran VK, Murali R, Krishna M, Shamala A, Yalamalli M, Kumar AV. Knowledge, attitude, and practice of needle stick and sharps injuries among dental professionals of Bangalore, India. Journal of International Society of Preventive & Community Dentistry **2015**; 5(5): 406-12.
.69 Rampal L, Zakaria R, Sook LW, Zain AM. Needle stick and sharps injuries and factors associated among health care workers in a Malaysian hospital. Eur J Soc Sci **2010**; 13(3): 354-62.
.70 Reddy S, Joseph V, Liu Z, Straker T. Characteristics of sharp injuries in anaesthesia providers in New York state: A cross-sectional study. J Clin Diagn Res **2018**; 12(1.10-6 :(2
.71 Salmanzadeh S, Rahimi Z, Goshtasbipour M, Meripoor M. The Prevalence of Needle-Stick Injuries among Healthcare Workers in Dasht-e-Azadegan, Southern West of Iran. International Journal of Pharmaceutical Research and Allied Sciences **2016**; 5(2.22-417 :(
.72 Seng M, Lim JW, Sng J, Kong WY, Koh D. Incidence of needlestick injuries among medical students after implementation of preventive training. Singapore Med J **2013**; 54(9): 496-500.
.73 Seng M, Sng GKJ, Zhao X, Venkatachalam I, Salmon S, Fisher D. Needlestick injuries at a tertiary teaching hospital in Singapore. Epidemiol Infect **2016**; 144(12): 2546-51.
.74 Shah SF, Bener A, Al-Kaabi S, Al Khal AL, Samson S. The epidemiology of needle stick injuries among health care workers in a newly developed country. Saf Sci **2006**; 44(5): 387-94.
.75 Shiao JS, McLaws ML, Huang KY, Guo YL. Student nurses in Taiwan at high risk for needlestick injuries. Annals of epidemiology **2002**; 12(3): 197-201.

.76 Shiva F, Sanaei A, Shamshiri AR, Ghotbi F. Survey of needle-stick injuries in paediatric health personnel of 5 university hospitals in Tehran. JPMA The Journal of the Pakistan Medical Association **2011**; 61(2): 127-31.
.77 Small L, Pretorius L, Walters A, Ackerman MJ. A surveillance of needle-stick injuries amongst student nurses at the University of Namibia. Health SA Gesondheid **2011**; 16.(1)
.78 Smith DR, Smyth W, Leggat PA, Wang RS. Needlestick and sharps injuries among nurses in a tropical Australian hospital. International journal of nursing practice **2006**; 12.7-71 :(2)
.79 Smith DR, Leggat PA. Needlestick and sharps injuries among nursing students. Journal of advanced nursing **2005**; 51(5): 449-55.
.80 Suliman M, Al Qadire M, Alazzam M, Aloush S, Alsaraireh A, Alsaraireh FA. Students nurses' knowledge and prevalence of Needle Stick Injury in Jordan. Nurse education today **2018**; 60: 23-7.
.81 Swe KMM, Zin T, Bhardwaj A, Abas ABL, Barua A. The prevalence of needle sticks injury among medical students in Melaka, Malaysia, a cross sectional study. Eur J Sci Res **2012** ; .20-214 :(2)71
.82 Trinkoff AM, Le R, Geiger-Brown J, Lipscomb J. Work schedule, needle use, and needlestick injuries among registered nurses. Infection control and hospital epidemiology **2007**; 28(2): 156-64.
.83 Wang S, Yao L, Li S, Liu Y, Wang H, Sun Y .Sharps injuries and job burnout: A cross-sectional study among nurses in China. Nurs Health Sci **2012**; 14(3): 332-8.
.84 Wicker S, Jung J, Allwinn R, Gottschalk R, Rabenau HF. Prevalence and prevention of needlestick injuries among health care workers in a German university hospital. Int Arch Occup Environ Health **2008**; 81(3): 347-54.
.85 Boal WL, Leiss JK, Sousa S, Lyden JT, Li J, Jagger J. The national study to prevent blood exposure in paramedics: exposure reporting. American journal of industrial medicine **2008**; 51(3): 213-22.
.86 Xujun Z, Yue G, Mengjing C, Lorann S, Huiyun X. Needlestick and sharps injuries among nurses at a teaching hospital in China. Work **2015**; 63(5): 219-25.
.87 Yao WX, Yang B, Yao C, et al. Needlestick injuries among nursing students in China. Nurse Educ Today **2010**; 30(5): 435-7.
